# Ablative Radiotherapy in Prostate Cancer: Stereotactic Body Radiotherapy and High Dose Rate Brachytherapy

**DOI:** 10.3390/cancers12123606

**Published:** 2020-12-02

**Authors:** Ting Martin Ma, Oscar Lilleby, Wolfgang A. Lilleby, Amar U. Kishan

**Affiliations:** 1 Department of Radiation Oncology, University of California, Los Angeles, CA 90095, USA; TMa@mednet.ucla.edu; 2 Faculty of Medicine, University of Copenhagen, 2200 Copenhagen, Denmark; tqw792@alumni.ku.dk; 3 Department of Oncology, Oslo University Hospital, 0424 Oslo, Norway; WLL@ous-hf.no; 4 Department of Urology, University of California, Los Angeles, CA 90095, USA

**Keywords:** ablative radiotherapy, prostate cancer, stereotactic body radiotherapy (SBRT), high-dose-rate brachytherapy (HDRBT), biochemical recurrent free survival (bRFS), toxicity

## Abstract

**Simple Summary:**

Radiation therapy is a standard of care treatment option for men with localized prostate cancer. Over the years, various radiation delivery modalities have contributed to the increased precision of radiation, employing radiobiological insights to shorten the overall treatment time with hypofractionation, while improving oncological control without increasing toxicities. Here, we discuss and compare two ablative radiation modalities, stereotactic body radiation therapy (SBRT) and high-dose-rate brachytherapy (HDRBT), in terms of oncological control, dose/fractionation and toxicities in men with localized prostate cancer. This review will highlight the levels of evidence available to support either modality as a monotherapy, will summarize safety and efficacy, help clinicians gain a deeper understanding of the safety and efficacy profiles of these two modalities, and highlight ongoing research efforts to address many unanswered questions regarding ablative prostate radiation.

**Abstract:**

Prostate cancer (PCa) is the most common noncutaneous solid organ malignancy among men worldwide. Radiation therapy is a standard of care treatment option that has historically been delivered in the form of small daily doses of radiation over the span of multiple weeks. PCa appears to have a unique sensitivity to higher doses of radiation per fraction, rendering it susceptible to abbreviated forms of treatment. Stereotactic body radiation therapy (SBRT) and high-dose-rate brachytherapy (HDRBT) are both modern radiation modalities that allow the precise delivery of ablative doses of radiation to the prostate while maximally sparing sensitive surrounding normal structures. In this review, we highlight the evidence regarding the radiobiology, oncological outcomes, toxicity and dose/fractionation schemes of SBRT and HDRBT monotherapy in men with low-and intermediate-risk PCa.

## 1. Introduction

Most patients diagnosed with prostate cancer (PCa) in the developed world present with clinically localized disease, and the majority have low- or intermediate-risk disease as defined by the National Comprehensive Cancer Network (NCCN) [1]. External beam radiotherapy (EBRT) is a standard of care option for all patients with localized prostate cancer. Conventionally fractionated EBRT (CF-RT) consists of daily doses or fractions (1.8–2.0 Gy each) delivered over 39–45 treatment sessions. Considerable data suggest that PCa has a low alpha/beta ratio ranging from 1.5 to 3.1 [2], implying a preferential response to higher doses per fraction compared to most other tumors and normal tissues [3]. This has motivated multiple clinical trials investigating higher dose-per-fraction regimens. Moderate hypofractionation (MHF-RT) regimens, which deliver 2.4–3.4 Gy per day over 20–30 treatment sessions, have been studied extensively, with three non-inferiority randomized clinical trials demonstrating the efficacy and safety of this approach [4,5,6] and one superiority trial showing improved outcomes without worse toxicity [7], establishing it as the preferred regimen for localized PCa [8]. Extreme or ultrahypofractionation radiotherapy (UHF-RT) regimens deliver ≥5 Gy per fraction; when advanced delivery techniques and ≤5 fractions are used, the approach is termed stereotactic body radiotherapy or stereotactic ablative radiotherapy (SBRT/SABR). UHF-RT has demonstrated oncologic non-inferiority compared with CF-RT in one randomized trial [9], and SBRT has demonstrated equivalent acute toxicity in another randomized trial [10]. Long-term data from pooled prospective studies [11], as well as a comprehensive meta-analysis [12], suggest a very favorable safety and toxicity profile for SBRT. As of 2020, UHF-RT is now listed as a standard radiation option for all patients with localized disease in the NCCN guidelines [13].

As an alternative to EBRT, prostate brachytherapy involves the placement of sealed radiation sources into the prostate. Radiation intensity follows the inverse square law; therefore, brachytherapy allows very high doses to be delivered intraprostatically, with a sharp dose gradient outside of the prostate establishing high conformity to the target volume. High-dose rate brachytherapy (HDRBT) is a form of brachytherapy in which high-activity radiation sources (e.g., iridium-192) are temporarily placed within the prostate, typically over two to three fractions. Radiobiologically, HDRBT leverages the low alpha/beta ratio of prostate cancer as well. HDRBT as a monotherapeutic option has been investigated in multiple institutional series [14,15]. As of 2020, it is considered a standard option for very low, low, and favorable intermediate risk disease per the NCCN guidelines [13].

In this review, we will comprehensively review the safety and efficacy profiles of SBRT and HDRBT for localized prostate cancer, beginning with a brief discussion of the unique radiobiological features of ablative radiotherapy. We will focus on monotherapy approaches for low- and intermediate-risk disease, as the preponderance of evidence exists in this setting. A detailed overview of the use of these modalities as monotherapies for high-risk disease, and their use as boost options in combination with EBRT, are beyond the scope of this review.

## 2. Radiobiology of Ablative Radiotherapy

There are several advantages of ablative radiotherapy (SBRT or HDRBT) compared to conventionally fractionated radiotherapy when treating prostate cancer. First of all, prostate cancer preferentially responses to higher doses per fraction of radiation compared to most other tumors and normal tissues given its low alpha/beta ratio (typically 1.5 to 3.1) [16]. Secondly, ablative RT is delivered over a much shorter period of time. Although this is not as dominant as a factor compared to fraction size for late-responding tissues, there is limited repopulation of the irradiated tissues with ablative radiation, bolstering its tumor-killing effects. Studies have shown that linear-quadratic model underestimates tumor control by SBRT [17], indicating that additional mechanisms may be at play in addition to DNA strand breaks and/or chromosome aberrations [18]. One of them may be significant vascular damage in tumors from SBRT, leading to indirect cell death [18,19]. In addition, Wang and colleagues demonstrated that ablative hypofractionated radiation therapy at ≥10 Gy per fraction enhances tumor-killing via preferential stimulation of necroptosis (i.e., programmed necrosis) [20]. In fact, a high expression of a key protein involved in activation of necroptosis (RIP3) is associated with improved local control and progression-free survival in patients with non-small cell lung cancer. Last but not least, recent studies have demonstrated an immuno-modulatory effect of ablative radiotherapy, which is a very active area of ongoing investigation. Using a 16-gene tumor inflammation signature, Kean et al. demonstrated that most localized PCa are “cold” (low immune activation state) tumors pre-HDRBT while HDRBT converted 80% of these “cold” tumors into an “intermediate” or “hot” tumor [21]. Radiation depending on the fraction size increases peptide repertoire, enhances MHC class I expression, and facilitates killing by cytotoxic T lymphocytes [22,23]. One area under intense research is the possible synergy between ablative radiation and immunotherapy [24].

However, it should also be noted that the advantage of a large fraction size of ablative radiation may potentially be partially offset by the hypoxic clonogens within the tumor. PCa ranks high among the malignancies in which hypoxia plays a major role in treatment resistance and metastases [25,26]. In fact, hypoxia-associated gene expression has been correlated with Gleason score [27] and early biochemical relapse after radiotherapy and local recurrence [28]. As the alpha/beta ratio of hypoxic tumor cells is higher [29], increasing the fraction size (for a similar, total, biologically effective dose (BED)) will only have a modest influence on the control of those tumors containing significant numbers of hypoxic clonogens [16].

## 3. SBRT as Monotherapy

The first patient receiving modern SBRT was treated in 2000 as part of a prospective single-arm trial based at the Virginia Mason Hospital [30]. Subsequently, SBRT was studied in a variety of prospective, single-institution and multi-institutional phase II trials. Concurrently to these studies of SBRT, a large non-inferiority randomized trial, the HYPO-RT-PC trial [9], was initiated in 2005 and compared a seven-fraction UHF-RT against CF-RT; the majority of patients were treated with older radiation techniques. Finally, in 2012, the PACE-B randomized trial comparing modern SBRT against modern MHF-RT and CF-RT was launched [10]. Long-term results from HYPO-RT-PC and pooled results from multiple single-arm phase II studies [11], as well as early results from PACE-B, were published in 2019, leading to SBRT becoming an accepted standard of care option in 2020 for low- and intermediate-risk prostate cancer. In this section, we will provide a detailed overview of selected prospective studies evaluating UHF-RT and specifically SBRT. The selected monotherapy series are summarized in Table 1.

### 3.1. Prospective Evidence

The HYPO-RT-PC trial [9] compared 5-year failure-free survival (FFS) rates between patients with intermediate- and high-risk disease treated with CF-RT or UHF-RT [9]. The trial, which enrolled 1200 men across 12 centers from 2005 to 2015, was powered to demonstrate the non-inferiority of UHF-RT. Androgen deprivation therapy (ADT) was not used with EBRT on either arm, although the study population was comprised of patients with intermediate-risk (89%) and high-risk (11%) disease. With a median follow-up time of 5 years, the 5-year FFS rates were 84% in both groups (adjusted hazard ratio 1.002 (95% CI 0.758−1.325; *p* = 0.99)). Acute patient-reported outcomes on the Prostate Cancer Symptom Scale (PCSS) urinary and bowel scales were significantly worse with UHF-RT at the end of treatment, with urinary scores remaining significantly worse three months later. A slight difference was notable one-year post-treatment for both urinary PCSS scores and physician-scored grade ≥ 2 genitourinary (GU) toxicity on the Radiotherapy Therapy Oncology Group (RTOG) scale. However, the 5-year cumulative incidence of grade ≥ 2 GU and gastrointestinal (GI) toxicities were similar in both arms. No differences in the preservation of erectile function were seen. Overall, these results confirmed the oncologic non-inferiority of UHF-RT compared with CF-RT, at least in the intermediate term, establishing UHF-RT as a valuable standard of care option for prostate cancer.

Importantly, the radiation planning technique used for 80% of patients was three-dimensional conformal radiotherapy, rather than the more modern intensity modulated radiotherapy (IMRT). The latter may be associated with lower absolute rates of toxicity [31]. Additionally, while 90% of patients had implanted fiducial markers to help track the target and thereby mitigate the impact of prostate motion between fractions, the planning margins used were 7 mm isotropically, which would be considered large by contemporary standards. Thus, the absolute rates of toxicity in both arms of the HYPO-RT-PC trial are likely higher than what would be expected with modern techniques.

The PACE-B trial directly compared modern SBRT (36.25 Gy in five fractions of 7.25 Gy each; delivered consecutively or over the span of generally 2 weeks) with a control arm that allowed either CF-RT (78 Gy in 39 fractions of 2 Gy each; 31% of patients) or MHF-RT (62 Gy in 20 fractions of 3.1 Gy each; 69% of patients). This trial, which enrolled 874 men with low- and intermediate-risk PCa (Gleason 4 + 3 excluded) across 37 centers between 2012 and 2018, was designed to demonstrate the non-inferiority of SBRT with respect to freedom from biochemical or clinical failure at five years. The authors recently reported the results of a per-protocol, pre-specified substudy evaluating acute physician-scored toxicity on the RTOG scale [10]. The authors found that the cumulative rates of worst RTOG grade ≥ 2 GI toxicities exceeding baseline were 9.3% vs. 13.2% (SBRT vs. control arm), while for grade ≥ 2 GU toxicities, the rates were 20.2% vs. 26.8%. These typically occurred 4–6 weeks after the end of treatment, and by 12 weeks the rates dropped precipitously. No significant differences in GU or GI toxicities were identified at any timepoint by the RTOG scale. Similarly, no differences were found in the secondary endpoints of Expanded Prostate Cancer Index Composite (EPIC) bowel, urinary, and sexual bother, or worse acute grade ≥ 2 GU toxicity exceeding baseline on the Common Terminology Criteria for Adverse Events (CTCAE) scale. However, patients treated with SBRT did have significantly worse acute CTCAE grade ≥ 2 GI toxicity exceeding baseline (15.2% vs. 8%, *p* = 0.011), though this difference disappeared by 12 weeks. Overall, the PACE-B trial provides high-level evidence that the acute urinary and bowel outcomes with UHF-RT in HYPO-RT-PC may not be seen with more modern SBRT techniques.

Indeed, when directly comparing acute grade ≥ 2 RTOG GU toxicity rates, the rates were lower in PACE-B than HYPO-RT-PC (20.2% vs. 28%). This might be reflective of the use of IMRT and possibly reduced margins (4–5 mm isotropically, except 3–5 mm posteriorly). Further room for improvement may exist, however. Only 73% of patients treated with SBRT on PACE-B had implanted fiducial markers, and only 41.7% of patients had motion monitoring during treatment. Additionally, longer treatment intervals between SBRT fractions have been associated with decreased toxicity in prior studies [11,32] and an acute increase in physician-reported GI toxicity has been noted with moderate hypofractionation [8]. Daily fractionation was used for 20.7% of patients treated with SBRT on the PACE-B, and 69% of patients treated on the control arm received a moderately hypofractionated regimen. Thus, it may be important to dichotomize the control and experimental treatment arm by fractionation schedule, particularly when evaluating the impact of treatment time and fractionation for patients treated with SBRT. This will be critical in determining the optimal patients for treatment with SBRT. Of note, patients treated on HYPRO-RT-PC and PACE-B trials did not receive ADT; in the former case, this was due to lack of information regarding the importance of ADT at the time of trial design, while in the latter case it was due to the overall favorable risk nature of patients enrolled on the study.

The longest-term outcome data for modern SBRT comes from a pooled consortium study that included data from 12 single-arm phase II studies that together enrolled 2142 patients between 2000–2012 [11]. The median follow-up was 6.9 years. The trials generally enrolled patients with low-risk (55.3%) and favorable intermediate-risk disease (32.3%), though a minority had unfavorable intermediate-risk disease (12.4%). ADT was used in a minority of patients. Regimens ranged from 38 Gy in four fractions of 9.5 Gy each to 40 Gy in five fractions of 8 Gy each. The cumulative incidences of late grade ≥ 3 or higher toxic events by either RTOG or CTCAE scales (as defined by the individual studies included) were evaluated based on central review. With a median follow-up of 6.9 years, the seven-year cumulative incidence of late grade ≥ 3 GU toxicities was 2.4%, and the rate of grade ≥ 3 GItoxicities was 0.4%. The seven-year cumulative incidence biochemical recurrence was 4.5% for low-risk disease, 8.6% for favorable intermediate-risk disease, and 14.9% for unfavorable intermediate-risk disease. This compares favorably to the Conventional Versus Hypofractionated High-Dose Intensity Modulated Radiation Therapy for Prostate Cancer (CHHiP) trial, which demonstrated that 4 weeks of hypofractionated RT (HF-RT) was non-inferior regarding BCR and toxicity compared with 8 weeks of CF-RT. In the CHHiP trial, 8-year biochemical recurrence-free survival (bRFS) for men with low-, intermediate-, and high-risk disease was as 83.7% (comprised of 15% low-, 73% intermediate-, and 12% high-risk patients) [33], compared with 7-year bRFS rates of 95.5% and 89.9% with SBRT for low- and intermediate-risk disease in the consortium study. In the CHHiP trial, 97% of participants received 4–6 months of ADT via either medical castration or a direct anti-androgen [34]. These results are consistent with a systematic review and study-level meta-analysis [12], which reports a 5- and 7-year bRFS rates of 95.3% and 93.7%, respectively, with favorable toxicity profile, in a cohort of patients with low-, intermediate- and high-risk patients. In both the pooled SBRT consortium [11] and the meta-analysis [12], ADT use was not significantly associated with bRFS, though such analyses were likely underpowered and susceptible to selection biases.

Though not randomized evidence, this report provides prospectively collected multi-institutional data in a large cohort of patients treated with SBRT. Of note, the patients included in this report received treatments with protocols most reflective of modern SBRT delivery, with tighter margins (2–5 mm isotropically), 100% implanted fiducial markers and 88% real-time motion management during treatment. Indeed, the low absolute rates of grade ≥ 3 toxicity seen at seven years (over three-fold less than in the HYPO-RT-PC trial) may be secondary to these technological improvements.

### 3.2. SBRT Dose

As the evidence supporting SBRT as a standard of care monotherapy option has grown, further questions involving the technical aspects of treatments, such as dose and fractionation, have emerged. While a comprehensive overview of all studies is beyond the scope of this study, we will briefly review the emerging data regarding SBRT dosing and fractionation.

The fundamental premise behind dose-escalation is that locally recurrent or persistent disease may ultimately seed distant metastases. As such, dose escalation efforts are geared to optimize local control, but may not directly address micrometastatic regional or distant disease that exists on presentation. It is generally acknowledged that, particularly for aggressive disease, metastatic failure may be a more common mode of relapse; however, local relapse precedes a large proportion of metastatic failures at longer-term follow-up [35,36]. Dose escalation has been widely studied in the context of CF-RT. Of the multiple randomized trials that have been published, only one suggested an improvement in prostate cancer-specific survival [37], and only two suggested an improvement in the incidence of metastasis [38,39]. Nearly all studies have, however, shown an improvement in biochemical control with dose-escalation—albeit at the cost of increased GI, if not also GU, toxicity.

If we assume that the alpha/beta ratio of prostate cancer is indeed low (on the order of 2–3), an SBRT dose of 36.25 Gy in five fractions would have an equivalent dose in 2-Gy fractions (EQD2) ranging from 74.3–83.8 Gy. Thus, this dose would be roughly commensurate with modern, dose-escalated CF-RT, and no randomized trials have evaluated doses in the context of UHF-RT. The aforementioned study-level meta-analysis found that increasing BED was significantly associated with improved bRFS [12]. In contrast, a subset analysis of the pooled SBRT consortium failed to reveal an association between EQD_2_ and time to BCR for any risk group [11]. A detailed comparison of bRFS in 1908 patients after 35 Gy/five fractions, 36.25 Gy/five fractions, 40 Gy/five fractions, and 38 Gy/four fractions found a difference in prostate ablation, without a clear difference in bRFS at 5-years [40]. However, at a median follow-up of 72.3 months, treatment with 40 Gy/five fractions was associated with improved long-term bRFS when compared against all other doses. The decoupling of prostate tumor ablation and bRFS between 38 Gy/four fractions and 40 Gy/five fractions could be explained by extraprostatic failures presenting a dominant pattern of failure after SBRT at this dose level, though the lack of a significant difference between 38 Gy/four fractions and lower SBRT doses is more difficult to explain. The general finding of increased local control/prostate tumor ablation by escalating dose up to 40 Gy is in accordance with the results of a prospective dose-escalation trial from Memorial Sloan Kettering Cancer Center (MSKCC) of 136 patients who received SBRT at doses ranging from 32.5 Gy/five fractions to 40 Gy/five fractions [41]. In that trial, significantly fewer positive biopsies were seen two years after radiotherapy with escalating dose. Improved PSA response with 40 Gy/five fractions vs. 35 Gy/five fractions was also seen in a comparison of two single-arm prospective studies, although no difference in bRFS was found [42]. A plateau to the dose response was also suggested by a recent modeling study [43]. It is acknowledged that there are few data exploring doses above 40 Gy applied with UHF-RT [44,45].

Given the unclear benefit for dose escalation above 40 Gy, the cost must be considered. In one study comparing two prospective five-fraction SBRT studies of 40 vs. 35 Gy [46], patients receiving 40-Gy SBRT experienced greater grade ≥ 2 cumulative GU (24.2% vs. 5%) and GI (26.2% vs. 7.6%) toxicities by the RTOG scale compared to those receiving 35-Gy. Notably, the 40-Gy arm also utilized larger PTV margins (5 vs. 4 mm). In the aforementioned prospective MSKCC study [41], the 40-Gy dose cohort had significantly higher International Prostate Symptom Score (IPSS) scores at 12 months compared to lower dose cohorts. At 24 months, however, the increase in IPSS was only marginally significant compared to the 32.5-Gy group but not others. Finally, at 36 months no significant differences were observed across the various dose cohorts. An overall decrease in erectile function was also seen at 24 months across the board with no significant difference among different dose levels. Taken together, the results suggest that dose escalation may be associated with worse physician-scored toxicity and IPSS, though the effect on IPSS dissipates within 3 years.

### 3.3. SBRT Fractionation

The earliest studies of prostate SBRT treated patients on consecutive days (QD) [30,47]. Early results from the initial cohort of patients treated on the Stanford trial demonstrated an unexpectedly high rate of grade ≥ 2 rectal toxicity, motivating an every-other-day (QOD) schedule. Specifically, 0% vs. 38% of patients reported “moderate” or “big problem” in terms of rectal quality of life with QOD vs. QD fractionation [47]. Subsequently, treating QOD became the *de facto* standard in many studies. A subset analysis of the pooled SBRT consortium showed that QOD treatment was associated with lower odds of acute grade ≥ 2 toxicity than QD fractionation [11]. The PATRIOT trial [32] randomized patients to 40 Gy in five fractions delivered once weekly (QW) or QOD and demonstrated superior acute bowel and urinary QOL with QW treatments. At a median follow-up of 62 months [48], no differences in late toxicity, QOL, BCR were noted, although more patients on the QW arm received salvage ADT (0 vs. four patients). Two-fraction and single-fraction SBRT have been studied in the TWOSTAR and ONE SHOT trials, respectively; longer-term follow-up is awaited for both [49,50].

## 4. HDRBT as Monotherapy

### 4.1. HDRBT vs. LDRBT

Brachytherapy can be delivered either via a permanent low-dose-rate seed implant (LDRBT), or via HDRBT. LDRBT provides a continuous, ever-decreasing dose of radiation over time, while HDRBT delivers large doses per fraction. Potential advantages over HDRBT, beyond harnessing the putative low alpha/beta ratio of prostate cancer, include the fact that no radioactivity remains in the patient once treatment is completed and the fact that HDRBT provides tight control over dose delivery. HDRBT is less susceptible to issues related to prostate edema and seed migration that might complicate dosimetry following LDRBT. The larger dose per fraction, if delivered as a single fraction, may even induce transcriptional changes in the tumor genome, enhancing sensitivity to subsequent radiation exposure [51].

Current evidence suggests that HDRBT affords better tolerability with similar oncological control compared to LDRBT. Hathout et al. performed a phase II randomized trial comparing HDRBT (19 Gy × 1) with LDRBT (144 Gy ^125^I) in 31 men with favorable risk prostate cancer [52]. IPSS and EPIC urinary irritative scores were better with HDRBT at 1, 3, 6, and 12 months after treatment. Time to IPSS normalization was significantly shorter in the HDRBT group (two months) versus the LDRBT group (six months). There were no significant differences in the EPIC urinary incontinence, sexual, or bowel habit scores between the two groups at any measured timepoints. Similar findings have been reported in retrospective comparisons. Martinez et al. reported less acute GU and GI toxicities, as well as long-term GU toxicities with HDRBT than LDRBT [53], with equivalent 5-year biochemical control rates. Another retrospective monotherapy comparison of HDRBT and LDRBT with or without EBRT [54] showed similar 5-year bRFS between the two modalities (92.9% vs. 95.6%). LDRBT showed a higher incidence of acute grade ≥ 2 GU toxicity than that of HDRBT, but no difference in accumulated incidence of late grade ≥ 2 GU and GI toxicity. Interestingly, LDRBT appears to achieve a greater degree of prostatic ablation compared to HDRBT with lower nPSA. However, this does not appear to translate into a meaningful difference in oncological outcomes (discussed in detail in Section 5 below) [40]. This offers another example of the decoupling of the extent of prostate tumor ablation and bRFS beyond a certain dose level when there is no difference in bRFS despite a steeper PSA decay slope and lower nadir PSA (nPSA).

### 4.2. Evidence for HDRBT Monotherapy

While no randomized trials have compared HDRBT monotherapy directly to EBRT alone, the use of brachytherapy in general as a monotherapy for intermediate risk disease is supported by the RTOG 0232 trial, which has been presented in abstract form [55]. This trial randomized 588 men with intermediate risk disease to LDRBT monotherapy versus EBRT + LDRBT. Freedom from progression was similar at a median follow-up of 6.7 years, with 5-year progression free survival rates of 86% with LDRBT versus 85% with EBRT + LDRBT. Toxicity in both groups was limited, but there was a shift towards higher grade toxicities, as well as poorer patient-reported outcomes at 2 years in the EBRT + LDRBT arm than the LDRBT monotherapy arm. [56]. The authors conclude that men with intermediate-risk prostate cancer may safely receive brachytherapy alone. Given that LDRBT and HDRBT appear to have equivalent efficacy, HDRBT monotherapy would similarly appear to be an effective monotherapy option for men with intermediate-risk disease. Several single institution experiences provide specific data for HDRBT, as summarized in Table 2; 5-year BCR rates range from 90–95% [15,57,58,59,60,61,62]. Hauswald et al. reported the outcome of 448 men with low- and intermediate-risk PCa treated with HDRBT monotherapy with a median dose of 43.5 Gy in six fractions [15]. Local control at 10 years was excellent at 99.7% and 98.9% for low- and intermediate-risk patients, with 10-year bPFS of 98.9% and 95.2%, respectively. There were no significant differences in bPFS or OS between the two groups. No late-grade 3 to 4 rectal toxicities developed. Late-grade 3 to 4 genitourinary toxicity occurred in 4.9% (grade 3 in 4.7%). The single (0.2%) GU grade 4 toxicity was a urethral-rectal fistula that occurred after multiple transurethral resections. Similarly, Stouthos et al. reported excellent outcome of 450 men (198 low-, 135 intermediate- and 117 high-risk) treated with three single-fraction implants of 11.5 Gy with an interfractional interval of 21 days [62]. Biochemical control at 5 years was excellent at 96.1%, 96.1% and 92.1% for low-, intermediate- and high-risk patients, respectively. Late-grade 2 and 3 GU adverse events occurred in 14.2% and 0.8% of patients, respectively. Late-grade 2 GI toxicity amounted to 0.4%, with no instances of grade 3 or greater adverse events.

### 4.3. HDRBT Fractionation

While early HDRBT regimens involved as many as six fractions, much effort has gone into exploring more abbreviated regimens [57]. The most common standard fractionation schemes are 9 Gy × 4 or 13.5 Gy × 2, either delivered with a single implant or over two implants spaced one week apart [57,63,64]. In one retrospective single-institution comparison of 7.25 Gy × 6 versus 13.5 Gy × 2, no differences in IPSS or SHIM were noted [65]. Multiple studies have subsequently investigated single-fraction regimens [59,60,61]. Hoskin et al. reported favorable results in a cohort of patients who received 19–20 Gy in a single fraction compared to two fractions of 13 Gy or three fractions of 10.5 Gy for intermediate-risk and high-risk patients, with a 4-year BFS rate of >90% and similar rates of late toxicity [58]. A national registry study from the UK found that HDRBT-BT delivering a single fraction of 19 Gy showed bRFS of 100% at 3 years in low risk patients, but only 86% and 75% at 3 year in intermediate- and high-risk groups, respectively [61]. A recently published Canadian phase II randomized trial compared a single fraction of 19 Gy to a two 13.5 Gy-fraction regimen in 170 men with low- (19%) and intermediate-risk (81%) PCa. With a median follow-up of 60 months [66], PSA decreased more quickly with a much lower median PSA at 5-years in the two-fraction arm (0.16 ng/mL vs. 0.65 ng/mL). The 5-year bDFS was also significantly higher at 95% in the two-fraction arm compared to 73.5% in the single-fraction arm. Late grade 2 and 3 urinary toxicity on the CTCAE scale were 45% and 1%, respectively, and were similar between arms. Late-grade 2 rectal toxicity was only seen in 1% of patients on either arm. Among six patients with local failure on the 19 Gy arm in whom baseline MRI was available, 15 of the recurrences were at the site of initial disease. While it is tempting to assume that further dose escalation may increase local control, the data to support this contention are mixed. Single-fraction escalation to 20.5 Gy as monotherapy produced a 6-year biochemical control rate of 82% in a cohort of low- and intermediate-risk patients, which is only marginally better than those previously reported with 19 Gy [67]. Alayed et al. compared single 19 Gy HDRBT with or without an additional MRI-guided focal dominant intraprostatic lesion (DIL) boost to at least 23 Gy in low- or intermediate-risk disease patients in a phase II clinical trial [68]. The median dose to the dominant lesion was 27.2 Gy. However, although MRI-guided focal boost was associated with low rates of toxicity, the 5-year cumulative BF rate was 31.3%, suggesting suboptimal control. The underperformance of single-fraction HDRBT might be related to relative radioresistance (compared with multifraction regimens) due to limited re-oxygenation and re-assortment [69,70]. The heterogeneity of alpha/beta ratios within a tumor may also result in the underperformance of single-fraction HDRBT, particularly at a dose of 19 Gy [66]. Further studies of single-fraction HDRBT should be conducted in prospective trials.

## 5. Comparisons between HDRBT and SBRT

Few studies have directly compared SBRT with HDRBT. Dosimetrically, HDRBT plans are highly heterogeneous, whereas SBRT can be homogeneous or heterogeneous (termed “virtual HDRBT”) [11,71]. In a pooled analysis with bRFS as the end point, there was no difference in outcome between SBRT and HDRBT [72].

Planning studies that have compared HDRBT with predominantly homogeneous SBRT plans have generally found improved urethral dosimetry with SBRT, and improved rectal dosimetry and higher intraprostatic doses with HDRBT [73,74,75]. To date, however, no study that directly compared monotherapy with either SBRT or HDRBT in patients not receiving concurrent ADT has identified any outcome differences. The largest such study is a multi-institutional report recently published by Levin-Epstein et al. [76], that explored bRFS and PSA kinetics among 3502 patients receiving SBRT, HDRBT, or LDRBT monotherapy. Seventeen-hundred-and-sixteen patients (49.0%) received SBRT, while 512 (14.6%) and 1274 (36.4%) received HDRBT and LDRBT, respectively. Most patients (63.5%) had low risk disease, while 24.8% and 11.7% had favorable and unfavorable intermediate-risk disease, respectively. Median nPSA and time to nPSA were 0.2 ng/mL at 44 months after SBRT and 0.1–0.2 ng/mL at 37 months after HDRBT, respectively. Forty-eight percent of patients achieved nPSA < 0.2 ng/mL after SBRT, versus 56% after HDRBT. After SBRT, 69% of men achieved a PSA < 0.4 ng/mL at 4 years, which is an early proxy marker for ultimate biochemical control [42,77], compared to 57% for men receiving HDRBT. No differences in bRFS were identified, with 6-year rates of ~97% for low-risk disease, ~93% for favorable intermediate-risk disease, and ~87.5–92.5% for unfavorable intermediate-risk disease after either SBRT or HDRBT. Interestingly, as mentioned above, LDRBT achieved the greatest degree of prostatic ablation compared to SBRT and HDRBT, as evident by the lowest nPSA.

From a patient perspective, those treated with HDRBT vs. SBRT were 7.4 times more likely to have regret, and patients treated with IMRT versus SBRT were 11.1 times more likely to have regret [78]. The majority of patients with regret wished they had elected active surveillance. Significantly more patients treated with SBRT were pleasantly surprised regarding their actual long-term toxicities compared with IMRT and HDRBT patients.

## 6. Future Directions for SBRT and HDRBT for Localized Disease

Several future avenues of research are emerging. First, numerous randomized clinical trials are underway to clarify the role of SBRT and HDRBT as monotherapy options in localized PCa. Three large randomized trials are ongoing to establish SBRT as the preferred standard option for localized disease (Table 3). The NRG GU-005 trial (NCT03367702) compares SBRT with moderately hypofractionated radiotherapy and is designed to confirm the superiority of SBRT. The PACE series trials (A-C) aim to assess whether SBRT (36.25 Gy in five fractions) offers a therapeutic benefit over prostatectomy or ordinary RT for patients with organ-confined prostate cancer (ISRCTN 17627211, NCT01584258). The PACE-A trial is a superiority trail comparing SBRT and prostatectomy in low- and intermediate-risk patients. PACE-C is comparing SBRT to ordinary radiotherapy with the primary end-point as freedom from biochemical or clinical failure at 5 years post-randomization. Toxicity assessment serves as one of the secondary endpoints. PACE-B has been described above; long-term results are eagerly awaited. For HDRBT monotherapy, a small, single-institution trial of 10 patients is designed to investigate the practical feasibility of attempting a larger randomized trial of HDRBT vs. SBRT (NCT04253483).

A second important area of research involves improving the technical delivery of radiation. The insertion of a hydrogel spacer between the rectum and the prostate has demonstrated improvements in various metrics in one randomized trial [79] and it remains unknown whether the use of such spacers would have a proportionately greater effect for patients treated with SBRT or HDRBT. Investigation of MRI-based treatment delivery platforms is also underway, which may allow enhanced visualization of the target, and therefore even tighter treatment margins [80]. The MIRAGE trial is a randomized phase III trial comparing MRI-guided SBRT with standard CT-guided SBRT for localized PCa, with the hypothesis that MRI-guided SBRT will lead to an improvement in the cumulative incidence of acute grade ≥ 2 GU toxicity when compared to CT-guided SBRT (NCT04384770).

Third, an exciting area of future research involves the use of precision medicine principles to guide the choice of fractionation and treatment intensification. Mounting evidence suggests that variations within a patient’s germline DNA might influence the severity of the adverse reactions they experience to various cancer therapies, including radiation [81,82]. Preliminary data suggest that certain germline single nucleotide polymorphism panels may predict for late GU toxicity after SBRT, while a distinct panel may predict for late GU toxicity after CF-RT [83]. Even if the absolute overall toxicity rates are similar for patients treated with SBRT or CF-RT, such panels may help to identify patients who are more likely to experience significant toxicity after one treatment regimen or the other. An improved understanding of the biological heterogeneity of prostate cancer and the development of prognostic and predictive biomarkers will help guide rational treatment intensification and de-escalation [84,85,86]. Together, advances in medical genetics may allow patients and physicians to obtain a roadmap of risk-adapted, individualized treatment options.

## 7. Conclusions

Overall, SBRT and HDRBT are both excellent monotherapy options for prostate cancer that deliver an ablative dose of radiation to cancer cells. The high oncologic efficacy of both modalities leverages the observation that prostate cancer exhibits an enhanced response to large fractions of radiotherapy. UHF-RT, which includes SBRT, has been demonstrated to be non-inferior to CF-RT in a large, prospective randomized trial, which, together with long-term data from other prospective, single-arm studies, established it as a standard of care option. Studies have shown unclear oncologic benefit for SBRT dose escalation above 40 Gy, with cost of increased toxicity. HDRBT, on the other hand, has never been prospectively compared with any EBRT regimen or with LDRBT. Regardless, the wealth of historical data demonstrating favorable oncologic outcomes have established it as a standard of care option. HDRBT affords better tolerability with similar oncological control compared to LDRBT. It has distinct dosimetric distributions and PSA kinetics compared to SBRT, but ultimately oncological control does not seem to be different based on current evidence. Further study is needed to optimize the therapeutic ratio by improving efficacy and minimizing toxicity.

## Figures and Tables

**Table 1:** Selected recent stereotactic body radiation therapy (SBRT) monotherapy series in low- and intermediate-risk patients.

Author	Year	N	Risk	FU-Med	Dose/fx	Outcome (bRFS)	Toxicity	ADT
Widmark ^†^ (HYPO-RT-PC trial)	2019	598	89% I 11% H	5 years	42.7 Gy/7 fx (3 days per week)	5-year bRFS: 84%	Acute RTOG grade ≥ 2 GU toxicity: 28%, acute RTOG grade ≥ 2 GI toxicity: 24%. 5-year grade ≥ 2 GU toxicity: 18%, grade ≥ 2 GI toxicity: 10%, grade ≥ 3 GU toxicity: 4.2%, grade ≥ 3 GI toxicity: 1.7%.	No ADT allowed
Brand ^†^ (PACE-B trial)	2019	433	8% L 92% I	12 weeks	36.25 Gy/5 fx (7.25 Gy/fx delivered consecutively (20.7%) or over the span of ~2 weeks (79.3%))	n/a	Acute RTOG grade ≥ 2 GU toxicity exceeding baseline: 20.2%. Acute RTOG grade ≥ 2 GI toxicity: 9.3%.	No ADT allowed
Kishan	2019	2142	55.3% L 32.3% I (F) 12.4% I (UF)	6.9 years	33.5–40.0 Gy in 4 to 5 fx (88% 5 fx).	7-year bRFS: 95.5% L; 91.4% F-I; 85.1% UF-I, 89.8% for all I.	Acute grade ≥ 2 GU toxicity: 9.6%, grade ≥ 2 GI toxicity: 3.4%, grade ≥ 3 GU toxicity: 0.6%, grade ≥ 3 GI toxicity: 0.09%. 7-year cumulative incidence of late grade ≥ 3 GU toxicity: 2.4%, grade ≥ 3 GI toxicity: 0.4%.	5.4% received concurrent ADT; 3.6% for L, 9.4% for I (U)
Levin-Epstein	2020	1908	50.0% L 30.9% I (F) 19.1% I (UF)	6.0 years	35 Gy/5 fx 36.25 Gy/5 fx 40 Gy/5 fx 38 Gy/4 fx	93.8% 93.3% 96.1% 91.1%	n/a	Upfront ADT excluded
Zelefsky	2019	136	33.1% L 44.1% I (F) 22.8% I (UF)	5.9 years 5.4 years 4.1 years 3.5 years	32.5 Gy/5 fx 35 Gy/5 fx 37.5 Gy/5 fx 40 Gy/5 fx	5-year bRFS: 85%, 94%, 100%, 100%; 2-year positive biopsy post-RT: 47.6%, 19.2%, 16.7%, 7.7%	Acute grade 2 GI toxicities: 0%, 2.9%, 2.8%, and 11.4%; no grade 3+. Acute grade 2 GU toxicities: 16.7%, 22.9%, 8.3%, and 17.1%; no grade 3+. Late grade 2 GU toxicities: 23.3%, 25.7%, 27.8%, and 31.4%; 1 late grade 3 urinary toxicity (urethral stricture) developed in the 40-Gy dose arm; no grade 4.	Neoadjuvant ADT excluded

Risk groups: L/I/H: low-risk/intermediate-risk/high-risk prostate cancer; F: favorable; UF: unfavorable; FU-med: median follow-up; bRFS: biochemical recurrence-free survival; ADT: androgen deprivation therapy; fx: fraction. ^†^ randomized controlled trial.

**Table 2:** Selected HDRBT monotherapy series.

Author	Year	N	Risk	FU-Med	Dose/fx	IMP	Outcome (bRFS)	Toxicity
Zamboglou	2013	718	L + I + H	4.4	34.5–38 Gy/3–4 fx	1–3	95% (L), 93% (I), 93% (H) @ 5 years	Acute: grade 3 GU: 5.4%; grade 3 GI: 0.2% Late: grade 3 GU: 3.5%, grade 4 GU: 0.3%; grade 3 GI: 1.6%, grade 4 GI: 0
Hauswald	2016	448	L + I	6.5	42–43.5 Gy/6 fx	2	99% (L), 95% (I) @ 10 years	Late: grade 3 GU: 4.7%, grade 4 GU: 0.2%; grade 3+ GI: 0
Jawad	2016	319	L + I	5.5	38 Gy/4 fx	1	97% (L + I) @ 5 years	Acute: grade 3 GU <1%, grade 4+ GU: 0; grade 3+ GI: 0 Late: grade 3 GU <1%, grade 4+ GU: 0; grade 3+ GI: 0
		79	L + I	3.5	24 Gy/2 fx	1–2	87% (L + I) @ 5 years	
		96	L + I	2.5	27 Gy/2 fx	1–2	90% (L + I) @ 5 years	
Yoshioka	2017	524	L + I + H	5.9	27 Gy/2 fx (13%), 45.5 Gy/7 fx (32%), 49 Gy/7 fx (28%), 54 Gy/9 fx (25%)	1	95% (L), 94% (I), 89% (H) @ 5 years	Acute: grade 3 GU: 0.2%; grade 3 GI: 0 Late: grade 3 GU: 1%, grade 3 GI: 0.2%; grade 4+ GU/GI: 0
Hoskin	2017	106	I + H	4.1	31.5 Gy/3 fx	1	94% (I + H) @ 4 years	Late: grade 3 GU: 2.5%; grade 3 GI: 0
		138	I + H	5.2	26 Gy/2 fx	1	93% (I + H) @ 4 years	Late: grade 3 GU: 1.0%; grade 3 GI: 0
		49	I + H	9	19–20 Gy/1 fx	1	91% (I + H) @ 4 years	Late: grade 3 GU: 2.2%; grade 3 GI: 0
Strouthos	2018	450	L + I + H	4.7	34.5 Gy/3 fx	3	96% (L), 96% (I), 92% (H) @ 5 years	Late: grade 2 GU: 14.2%, grade 3 GU: 0.8%; grade 2 GI: 0.4%, grade 3+ GI: 0
Siddiqui	2019	68	L + I	3.9	19 Gy/1 fx	1	79% (L), 75.2% (I) @ 5 years	Late: grade 2 GU: 14.7%, grade 3+ GU: 0; grade 2+ GI: 5.9%, grade 3 GI: 1.5%; grade 2+ sexual: 19.1%
Xu	2019	124	L + I	2.2	19 Gy/1 fx	1	90.3% (L + I) @ last f/u	Acute: grade 3+ GU: 0; grade 3+ GI: 0. Late: grade 2 GU: 60%, grade 3+ GU: 0; grade 2 sexual: 67%, grade 3 sexual: 7%
Tharmalingam	2020	441	L + I + H	2.2	19 Gy/1 fx	1	100% (L), 86% (I), 75% (H) @ 3 years	Acute: grade 2 GU: 12%; grade 2 GI: 3%; grade 3+ GI/GU: 0 Late: grade 3 GU: 0.4%; grade 3 GI: 0.4%; grade 4+ GU/GI: 0

Risk groups: L/I/H: low-risk/intermediate-risk/high-risk prostate cancer; FU-med: median follow-up in years; bRFS: biochemical recurrence-free survival; IMP: implant; fx: fraction; GU: genitourinary; GI: gastrointestinal.

**Table 3:** Selected list of important ongoing randomized SBRT and HDRBT trials.

Trial Registration No.	Acronym	Type	Patient Selection	Hypothesis/Objective	Interventions	Primary Outcome	Secondary Outcomes (Selected)	Recruiting Status
NCT03367702	NRG GU-005	Randomized phase III trial	Stage IIA-B PCa	SBRT is superior to hypofractionated IMRT in terms of GU/GI toxicities	Arm 1: hypofractionated IMRT (70 Gy/28 fx); Arm 2: SBRT (36.25 Gy/5 fx)	Incidence of patients-reported GU/GI toxicity; Disease Free Survival	BCF, distant metastasis, health-related QOL	Recruiting
NCT01584258	PACE	Randomized phase III trial	Localized low- and intermediate- risk (PACE-A, B), intermediate- and high-risk (PACE-C)	Compare oncological control and toxicities of prostatectomy vs. SBRT (PACE-A), SBRT to ordinary RT (PACE-B, C)	PACE-A: Surgical candidates: Arm 1: Laparoscopic Prostatectomy; Arm 2: SBRT (36.25 Gy/5 fx). PACE-B ^†^: Arm 1: CF-RT (78 Gy/59 fx) or MHF-RT (62 Gy/20 fx) Arm 2: SBRT (36.25 Gy/5 fx) PACE-C ^‡^: Arm 1: MHF-RT (60 Gy/20 fx) Arm 2: SBRT (36.25 Gy/5 fx)	Biochemical progression-free survival (For RT: Phoenix definition; for surgery: PSA > 0.2 ng/mL)	CTCAE and RTOG acute and late toxicity	Recruiting
NCT04384770	MIRAGE	Randomized phase III	Clinical localized adenocarcinoma of the prostate	MRI-guided SBRT offers better GU/GI toxicity profile over CT-guided SBRT	Arm 1: CT-guided SBRT (40 Gy/5 fx); Arm 2: MRI-guided SBRT (40 Gy/5 fx)	CTCAE acute grade ≥ 2 GU toxicity	CTACAE acute grade ≥ 2 GI toxicity. late GU/GI toxicity, QOL, BCRFS	Recruiting
NCT03424694	BRP2	Randomized phase II	Low- and intermediate-risk	Assess the safety and oncologic outcome of 1 vs. 2-fraction HDRBT on a single implant	Arm 1: 29 Gy/2 fx HDRBT, single implant Arm 2: 19.5 Gy/1 fx HDRBT	Acute RTOG GU and GI toxicity	OS, local PFS, distant PFS, biochemical failure, other toxicities	Active, not recruiting
NCT03426748	H17-02904	Randomized phase III	favorable and low-tier intermediate risk	Compare LDRBT vs. HDRBT	Arm 1: LDRBT Arm 2: HDRBT in 2 implants, 2 weeks apart (pts will undergo biopsies between the 2 fractions to assess tumor changes induced from the first fraction)	QOL in urinary domain (EPIC)	QOL in bowel and sexual domains, IPSS, acute and late toxicities, biochemical outcome	Recruiting
NCT02692105	n/a	Randomized phase III	Extensive favorable-risk and Intermediate-risk	Compare LDRBT vs. HDRBT	Arm 1: LDRBT Arm 2: HDRBT in 2 implants, 2 weeks apart (pts will undergo biopsies between the 2 fractions to assess tumor changes induced from the first fraction)	QOL in urinary domain (EPIC)	QOL in bowel and sexual domains, IPSS, acute and late toxicities, biochemical outcome	Recruiting
NCT02960087	n/a	Randomized phase II	Low- and intermediate-risk	Compare LDRBT vs. HDRBT	Arm 1: LDRBT with I-125 to a total dose of 144 Gy Arm 2: HDRBT: 27 Gy/2 fx	PSA at 48 months (<0.4 ng/mL)	DFS, acute and late toxicities, QOL, economic analysis	Recruiting

BCF: Biochemical failure; QOL: Quality of Life; OS: overall survival; DSS: disease-specific survival; LRF: locoregional failure; PFS: progression-free survival; CTCAE: Common Terminology Criteria for Adverse Events; EPIC: Expanded Prostate Cancer Index Composite; RTOG: Radiation Therapy Oncology Group; EBRT: external beam radiotherapy; HDRBT: high-dose-rate brachytherapy; LDRBT: low-dose-rate brachytherapy; PCa; prostate cancer; GU: genitourinary; GI: gastrointestinal; CF-RT: conventionally fractionated radiotherapy; HF-RT: hypofractionated radiotherapy; fx: fraction. ^†^ No ADT allowed; ^‡^ 6 months of ADT.
